# Nomogram model for predicting early recurrence for resectable pancreatic cancer: A multicenter study

**DOI:** 10.1097/MD.0000000000037440

**Published:** 2024-03-08

**Authors:** Quan Man, Huifang Pang, Yuexiang Liang, Shaofei Chang, Junjin Wang, Song Gao

**Affiliations:** a Department of Hepatobiliary and Pancreatic Surgery, Tongliao City Hospital, Tongliao, China; b Department of Pancreatic Cancer, Tianjin Medical University Cancer Institute and Hospital, National Clinical Research Center for Cancer, Key Laboratory of Cancer Prevention and Therapy, Tianjin’s Clinical Research Center for Cancer, Tianjin, China; c Department of Gastroenterology, Digestive Endoscopy Unit, Tongliao City Hospital, Tongliao, China; d Department of Gastrointestinal Oncology, the First Affiliated Hospital of Hainan Medical University, Haikou, China; e Department of Gastrointestinal Pancreatic Surgery, Shanxi Provincial People’s Hospital, Taiyuan, China.

**Keywords:** early recurrence, nomogram, pancreatic ductal adenocarcinoma, preoperative predictors, radical resection

## Abstract

Pancreatic cancer is a highly aggressive malignancy that is characterized by early metastasis, high recurrence, and therapy resistance. Early recurrence after surgery is one of the important reasons affecting the prognosis of pancreatic cancer. This study aimed to establish an accurate preoperative nomogram model for predicting early recurrence (ER) for resectable pancreatic adenocarcinoma. We retrospectively analyzed patients who underwent pancreatectomy for pancreatic ductal adenocarcinoma between January 2011 and December 2020. The training set consisted of 604 patients, while the validation set included 222 patients. Survival was estimated using Kaplan–Meier curves. The factors influencing early recurrence of resectable pancreatic cancer after surgery were investigated, then the predictive model for early recurrence was established, and subsequently the predictive model was validated based on the data of the validation group. The preoperative risk factors for ER included a Charlson age-comorbidity index ≥ 4 (odds ratio [OR]: 0.628), tumor size > 3.0 cm on computed tomography (OR: 0.628), presence of clinical symptoms (OR: 0.515), carbohydrate antigen 19-9 > 181.3 U/mL (OR 0.396), and carcinoembryonic antigen > 6.01 (OR: 0.440). The area under the curve (AUC) of the predictive model in the training group was 0.711 (95% confidence interval: 0.669–0.752), while it reached 0.730 (95% CI: 0.663–0.797) in the validation group. The predictive model may enable the prediction of the risk of postoperative ER in patients with resectable pancreatic ductal adenocarcinoma, thereby optimizing preoperative decision-making for effective treatment.

## 1. Introduction

In 2022, approximately 62210 patients in the US will be newly diagnosed with pancreatic cancer, equating to approximately 170 new cases each day.^[1]^ The incidence of pancreatic cancer is increasing at an annual rate of 0.5% to 1.0% and is projected to be the second leading cause of cancer-related deaths in the US by 2030.^[2]^ Pancreatic ductal adenocarcinoma (PDAC) accounts for most (90%) pancreatic neoplasms and has a 5-year survival rate of < 11%^[1]^; although it can be treated using surgical resection, only a small proportion of cases are detected at an early stage to allow this procedure. Moreover, even patients with resectable pancreatic cancer have unsatisfactory long-term outcomes, with a 5-year postoperative survival rate of just 20% to 30%, which is due to many of them relapsing even after curative resection, with a postoperative recurrence rate of 50% to 60%.^[3]^ The poor outcomes could be mainly attributed to the presence of occult tumor spread (which contributes to distant metastasis) as early as at the time of diagnosis, the persistence of tumor cells around the resection area (which is responsible for local recurrence) after radical tumor resection, and the lack of effective systemic therapies.^[4–6]^ Moreover, approximately 50% of patients experience recurrence within the first postoperative year, which is considered as early recurrence (ER).^[7]^

The high recurrence rate after tumor resection adversely affects the prognosis of pancreatic cancer; therefore, timely and accurate preoperative prediction of ER is crucial. Neoadjuvant or preoperative therapy can eradicate occult metastatic disease and make patients eligible for systemic therapy.^[8]^

Therefore, this study aimed to establish a clinical nomogram for predicting postoperative ER in patients with PDAC. These results may provide clinical guidance and improve survival outcomes in patients with resectable PDAC.

## 2. Methods

### 2.1. Ethics

This study was approved by the Ethical Review Committees of Tianjin Medical University Cancer Institute and Hospital, and conducted in accordance with the ethical guidelines of the Declaration of Helsinki. Informed consent was waived due to retrospective nature of the study.

### 2.2. Participants and setting

Patients in the model group were from the Department of Pancreatic Cancer, Tianjin Medical University Cancer Institute and Hospital, who underwent pancreatectomy for resectable PDAC from January 2011 to December 2020. In this dataset, the vast majority underwent open surgery; only 43 cases involved laparoscopic adjuvant surgery, and a mere 7 cases utilized da Vinci robotic surgery. Inclusion criteria were patients with potentially resectable PDAC located in either the head or body of the pancreas, encompassing those with both resectable and borderline resectable tumors. Resectable, border-resectable, and locally advanced pancreatic cancer is defined according to NCCN guideline version 2.2021.^[9]^ Exclusion criteria included applying neoadjuvant therapy, presence of synchronous distant metastasis at the time of resection, 3-month postoperative mortality, positive resection margins (R2), incomplete records, and a follow-up period of less than 12 months. Accordingly, we excluded 37 patients (2 patients with intraoperative metastases, 6 who died within 90 days, 6 who underwent preoperative chemotherapy, and 23 with incomplete follow-up data). Finally, we analyzed 604 cases, including 335 and 269 patients with and without ER, respectively (ER and late recurrence [LR] groups, respectively), and compared the clinicopathological parameters between the two groups. During the time of last follow-up, among 335(ER) of 604 cases had recurrd after a median RFS (recurrence-free survival) of 5.0 months (95% CI: 4.478–5.522), 269 (LR) patients had recurrd after a median RFS of 24.0 months (95% CI: 21.568–26.432).

The clinical data of patients with pancreatic ductal adenocarcinoma admitted to Shanxi Provincial People’s Hospital and the First Affiliated Hospital of Hainan Medical University from January 2020 to December 2021 were collected, and this group of patients was used as the verification group. The inclusion and exclusion criteria are the same as those of the model group.

In this study, ER was defined as recurrence within 12 months after surgery, and LR was defined as recurrence more than 12 months after surgery.

### 2.3. Data collection and follow-up

The preoperative demographics, clinicopathological characteristics, and treatment variables were extracted from a prospectively maintained institutional database. The preoperative Charlson age-comorbidity index (CACI) was calculated from available data as a frailty measure, with patients being dichotomized using a threshold of 4 points based on recent studies.^[10–12]^ We excluded CA19-9 levels measured when patients developed jaundice (total bilirubin level > 5 mg/dL) from the analysis. Further, we excluded patients with CA19-9 levels < 1.0 U/mL, which was considered negative for Lewis antigen. The main indicators were overall survival (OS), post-recurrence survival (PRS), and recurrence-free survival (RFS). RFS was calculated as the time from the date of pancreatectomy to the date of recurrence or last follow-up, whichever occurred first. Moreover, PRS was calculated as the time from the first postoperative recurrence to the final follow-up or death. Finally, OS was calculated as the time from radical resection to the final follow-up or death.

Follow-ups were performed at the outpatient clinic of our institution. The follow-up period was calculated from the surgery date to the date of death or last follow-up. The recurrence date was defined as the date when recurrence was shown in the first follow-up cross-sectional imaging study (computed tomography [CT], positron emission tomography [PET]/CT or magnetic resonance imaging [MRI]). Generally, follow-up CT or MRI for screening recurrence was performed every 90 days for the first 2 years, every 6 months for the next 3 years, and then annually for another 5 years. Biopsy was seldom performed when imaging findings indicated recurrence. Furthermore, in cases with elevated preoperative levels of carbohydrate antigen 19-9, levels were evaluated every three months. Recurrence sites were divided into the following five categories: “local only,” “liver only,” “lung only,” “multiple-site,” and “brain metastases or bone metastases.” Local recurrence was defined as a residual pancreas or operating bed recurrence. Distant recurrences are divided into three categories: liver recurrence alone, lung recurrence alone, and other less frequent sites. ‘‘Multiple distant’’ recurrence was indicated by both isolated local and distant recurrences being simultaneously observed in addition to recurrence occurring at multiple distant locations.

### 2.4. Statistical analysis

We performed between-group comparisons of clinicopathological features. A receiver operating characteristic (ROC) curve was constructed to estimate the optimal cutoff values of preoperative serum CA19-9 and CEA levels as risk factors for ER. To identify predictive factors for ER, we performed univariate and multivariate analyses of preoperative clinical data using a Cox proportional hazard regression model. Identified predictive factors were used to establish a nomogram.

Results are expressed as odds ratios (OR) with a corresponding 95% confidence interval (CI). Survival analysis was performed using the Kaplan–Meier method and between-group comparisons using the log-rank test. Statistical significance was set as *P* < .05.

The area under the curve (AUC) was calculated to evaluate the discrimination ability of the model on the training and validation populations. Calibration was evaluated using an unreliability test and a calibration curve.

All statistical analyses were performed using SPSS (version 26.0) and R (version 3.6.3) software.

## 3. Results

### 3.1. Patient cohort

**Figure 1.**
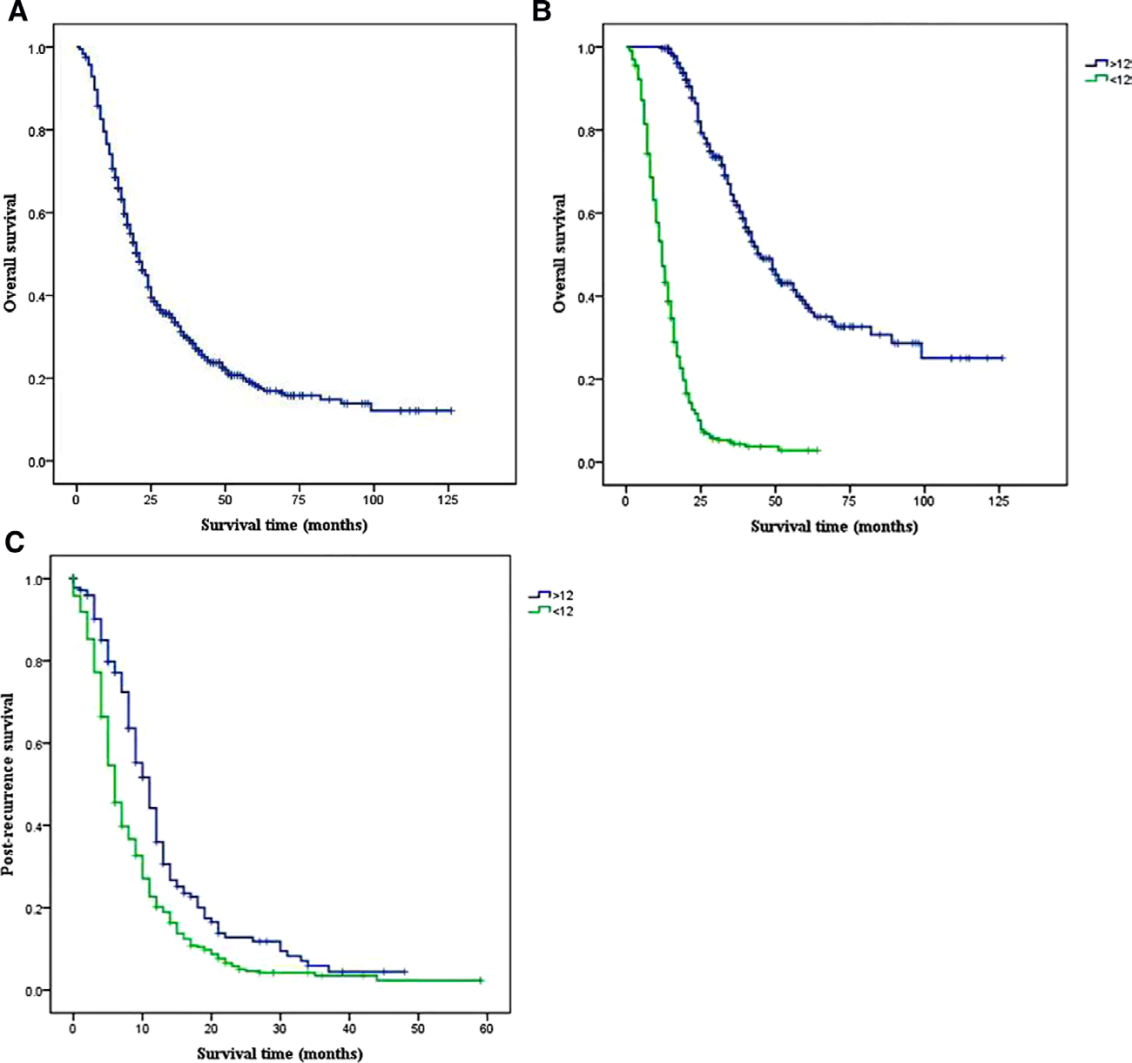
Kaplan–Meier survival curves of the overall patients. (A) ALL patients; (B) patients stratified according to recurrence time (recurrence within 12 months after surgery was considered as early recurrence (ER)); (C) Kaplan–Meier curves of postrecurrence survival according to the time of recurrence.

Table 1 summarizes patients’ demographic, clinicopathological, and treatment characteristics of the training and testing group. In the training and validation groups, the age, weight change, diabetes, pancreatic duct blockage and surgical modality were not statistically significant in early recurrence and late recurrence; While clinical symptoms, CCI, preoperative 199, CEA, and tumor size were statistically significant. In the training group, the median OS was 21 months; furthermore, in this group, 335 patients with ER and 269 patients with LR, respectively. The estimated 3- and 5-year survival rates were 30.3% and 18.3%, respectively (Fig. 1A). Moreover, there was a significant between-group difference in the median OS time (*P < *.001) (Fig. 1B), and the ER group had a shorter median PRS (6 months; 95% CI: 5.397–6.603) than did the LR group (11 months; 95% CI: 9.534–12.466) (Fig. 1C). The recurrence location was categorized as “local only,” “lung only,” “liver only,” “multiple-site,” and “other” in 157 (26.0%), 20 (3.3%), 203 (33.6%), 98 (16.2%), and 8 (1.3%) patients, respectively. Specifically, the “other” category encompasses instances such as brain metastases and bone metastases.

**Table 1:** Demographics, clinicopathologic, and treatment characteristics of included patients.

Variable	Training set			Testing set		
	Early recurrence < 12 mo (n=)	Late recurrece ≥ 12 mo (n=)	*P* value	Early recurrence < 12 mo (n=)	Late recurrece ≥ 12 mo (n=)	*P* value
Female, n (%)	133 (133/335)	119 (119/269)	.261	51 (51/222)	45 (45/222)	.151
Age, median (IQR)	61.07 (8.674)	59.41 (8.625)	.119	61.99 (8.519)	60.60 (7.630)	.211
Weight loss, n	190	138	.136	73	55	.590
Clinical symptoms						
Yes	246	158	.000	90	44	.001
No	89	111		40	48	
Diabetes						
Yes	87	76	.530	39	30	.679
No	248	193		91	62	
Charlson age-comorbidity index, n (%)						
<4 points	95	104	.008	36	44	.002
≥4 points	240	165		94	48	
Preoperative CA19-9 (U/mL)						
<181.3	143	176	.000	51	61	.000
≥181.3	191	93		79	31	
Preoperative CEA, U/L						
<6.01	228	222	.000	73	73	.000
≥6.01	105	45		57	19	
Preoperative CA 242 U/L						
<24.985	157	176	.000	59	59	.006
≥24.985	171	83		71	53	
Pancreatic duct dilation	335	269	.431	82	57	.865
Operation procedure, n (%)						
PD	220	181	.676	79	63	.239
DP	115	88		51	29	
CT tumour size, cm (SD)						
<3.0	148	150	.005	47	51	.004
≥3.0	187	119		83	41	
T-stage, n (%)						
1–2	236	221	.059	93	65	.886
3–4	99	48		37	27	

#### 3.1.1. PRS differences between ER and LR.

The median PRS was significantly longer in the LR group (11 months, 95% CI: 9.534–12.466) than in the ER group (6 months, 95% CI: 5.397–6.603) (*P < *.001; Fig. 1C). The 1- and 2-year PRS rates in the ER group were 20.2% and 5%, respectively, while the corresponding values in the LR group were 36% and 12.8%, respectively (both *P < *.001).

### 3.2. Risk factors for ER

Table 2 presents the results of the univariate analysis and multivariate logistic regression models with preoperative risk factors. Five preoperative variables were independently associated with ER: CACI score ≥ 4 (OR 0.639, 95% CI: 0.440–0.929, *P* = .019), tumor size > 3.0 cm on the last preoperative CT scan (OR: 0.686, 95% CI: 0.485–0.970, *P* = .033), preoperative carcinoembryonic antigen (CEA) > 6.01 U/mL (OR: 0.644, 95% CI: 0.418–0.988, *P* = .047), clinical symptoms (OR: 0.451, 95% CI: 0.310–0.654, *P* < .001), and preoperative CA 19–9 level > 181.3 U/mL (OR: 0.428, 95% CI: 0.304–0.602, *P* < .001). Results of the univariable analysis and multivariable logistic regression models for postoperative factors are presented in Table 3. Four postoperative risk factors independently correlated with early recurrence: postoperative CA 19-9 > 37 U/mL (OR 4.660, 95% CI: 3.085–7.040, *P* = .000), poor tumor differentiation grade (OR 1.939, 95% CI: 1.315–2.859, *P* = .001), microscopic lymphovascular invasion (OR 2.200, 95% CI: 1.459–3.318, *P* = .000), and adjuvant chemotherapy (OR 1.631, 95% CI: 1.063–2.503, *P* = .025).

**Table 2:** Univariate and multivariate analyses of predictive risk factors for ER in resectable pancreatic cancer.

Preoperative risk factors	Univariable		Multivariable	
	Odds ratio (95% CI)	*P* value	Odds ratio (95% CI)	*P* value
Age:<61years versus ≥ 61 years	0.730 (0.529–1.007)	.055		
Gender: female versus male	1.205 (0.870–1.668)	.261		
Weight loss	0.782 (0.556–1.080)	.136		
Clinical symptoms: yes or no	0.515 (0.365–0.726)	.000	0.451 (0.310–0.654)	.000
Diabetes: yes versus no	1.123 (0.783–1.610)	.530		
Expansion of the pancreatic duct	0.876 (0.630–1.218)	.431		
Charlson age-comorbidity index:	0.628 (0.446–0.884)	.008	0.639 (0.440–0.929)	.019
Tumor size	0.628 (0.455–0.867)	.005	0.686 (0.485–0.970)	.033
Tumor location	0.930 (0.662–1.307)	.676		
Preop CA 19-9	0.396 (0.284–0.551)	.000	0.428 (0.304–0.602)	.000
Preop CEA	0.440 (0.296–0.653)	.000	0.644 (0.418–0.988)	.047
Preop CA242	0.433 (0.308–0.608)	.000	0.689 (0.423–1.123)	.135

ER = early recurrence.

**Table 3:** Univariate and multivariate analyses of predictive risk factors for ER in resectable pancreatic cancer.

Postoperative risk factors	Univariable		Multivariable	
	Odds ratio (95% CI)	*P* value	Odds ratio (95% CI)	*P* value
Age:<61years versus ≥ 61 years	0.730 (0.529–1.007)	.055	–	–
Gender: female versus male	1.205 (0.870–1.668)	.261	–	–
Weight loss	0.782 (0.556–1.080)	.136	–	–
Clinical symptoms: yes or no	1.942 (1.378–2.737)	.000	2.318 (1.532–3.506)	.000
Diabetes: yes versus no	1.123 (0.783–1.610)	.530	–	–
Expansion of the pancreatic duct	0.876 (0.630–1.218)	.431	–	–
Charlson age-comorbidity index: ≥4 versus < 4	1.592 (1.132–2.241)	.008	1.672 (1.102–2.536)	.016
Postoperative CA 19–9 (>37 U/mL versus ≤ 37 U/mL)	4.352 (2.998–6.316)	.000	4.660 (3.085–7.040)	.000
Tumour differentiation well/moderate versus poor	2.307 (1.661–3.206)	.000	1.939 (1.315–2.859)	.001
Positive lymph nodes yes versus no	1.519 (1.049–2.199)	.027		
Micr. perineural invasion yes versus no	1.438 (1.018–2.032)	.039		
Micr. lymphovascular invasion, yes versus no	2.145 (1.547–2.975)	.000	2.200 (1.459–3.318)	.000
Chemotherapy versus no adjuvant	1.723 (1.177–2.523)	.005	1.631 (1.063–2.503)	.025

ER = early recurrence.

### 3.3. Establishment of the model and nomogram

**Figure 2.**
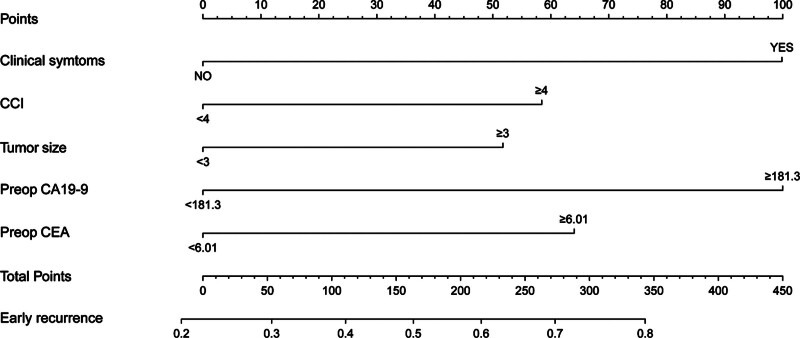
Nomogram for prediction of early recurrence after resection risk using preoperative parameters in resectable pancreatic ductal adenocarcinoma. CCI = Charlson age-comorbidity index, CEA = carcinoembryonic antigen.

**Figure 3.**
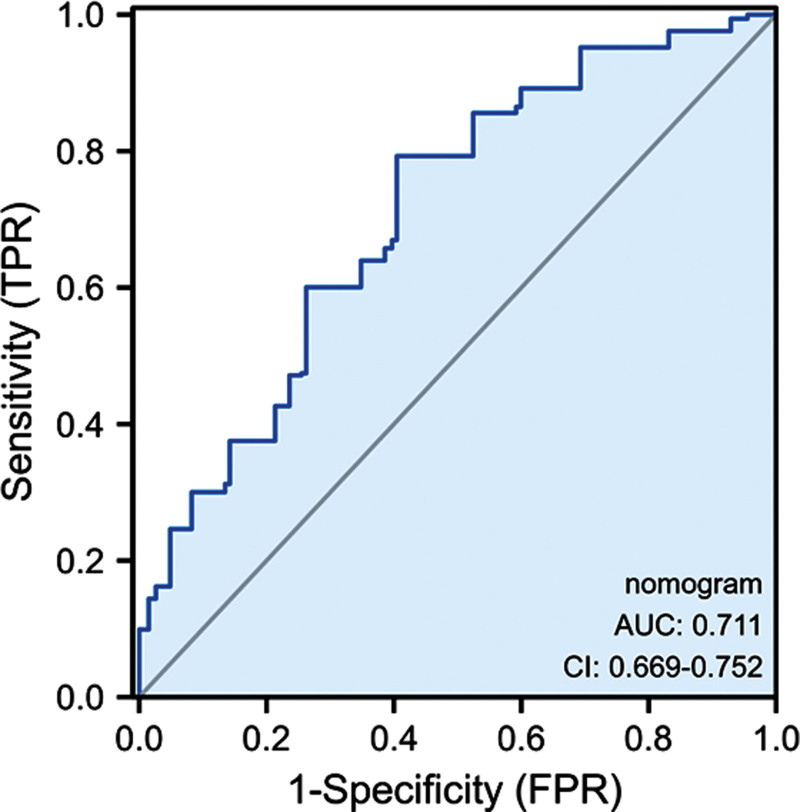
ROC curve of the established model. AUC = area under the curve, ROC = receiver operating characteristic.

**Figure 4.**
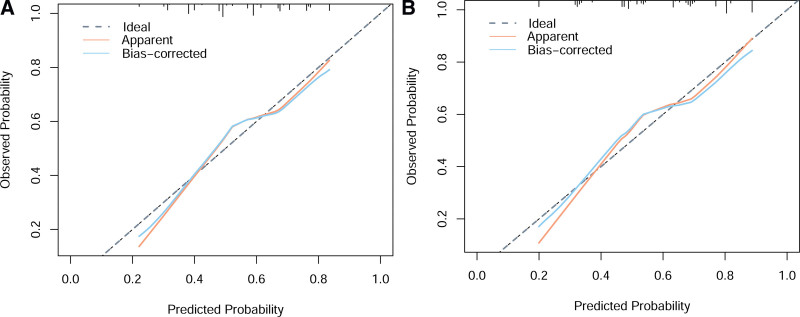
The calibration curves of the nomogram for the training set (A) and the testing set (B).

Based on the five aforementioned factors, we established a predictive model for ER in patients with resectable pancreatic cancer (Fig. 2). As shown in Figure 3, the AUC of the model was 0.711 (95% CI: 0.669–0.752). The sensitivity and specificity of the ROC curves were 0.793 and 0.596, respectively; furthermore, the optimal cutoff value was 158.87, and the calibration plot for ER showed good agreement between the predicted and observed rates (Fig. 4A). Finally, this model demonstrated a perfect fit.

In the testing set, the C-index was 0.682. Figure 4B shows the calibration curve; the goodness-of-fit value was 0.462. The sensitivity and specificity of the ROC curves were 0.892 and 0.520, respectively. Furthermore, the results of the testing set demonstrated that the prediction model exhibited good discrimination and prediction accuracy, thereby yielding favorable outcomes.

## 4. Discussion

ER in patients with PDAC is closely associated with survival outcomes; however, adjuvant therapy is correlated with a reduced likelihood of ER following pancreatectomy. Currently, reported preoperative risk factors for ER are inadequate for precisely identifying susceptible patients; thus, further study of new ER biomarkers is necessary. The challenge persists in choosing between surgery and neoadjuvant therapy for patients with ER of resectable PDAC. We contend that patients with ER who are ineligible for surgery should ideally undergo neoadjuvant therapy.

To our knowledge, there are no accepted preoperative prediction criteria for ER in patients with resectable pancreatic cancer. We constructed a clinical nomogram with good discrimination (OR: 0.711; 95% CI: 0.669–0.752) and calibration power for predicting ER in patients who underwent radical resection for PDAC. This model has potential clinical utility and may be used to inform treatment decisions and prompt adjustments of therapeutic strategies.

There remains no consensus regarding the definition of postoperative ER in patients with PDAC, which could be attributed to among-study differences in the research purposes and statistical methods. For example, several studies^[13–16]^ have defined ER as recurrence within six postoperative months. In contrast, based on RFS, Yang et al^[17]^ identified the optimal ER cutoff value as nine postoperative months. Furthermore, additional studies have established ER as a recurrence within 12 postoperative months.^[7,18–20]^ Specifically, Yamamoto et al^[18]^ based the optimal ER cutoff value on OS as 12 postoperative months, while Groot et al^[7]^ employed survival time after recurrence to define ER, positing that this metric better describes the biological behavior of the tumor than other definitions. These disparate reports indicate that a range of clinical factors may influence the definition of ER.

In our study, ER was defined as recurrence within 12 postoperative months; moreover, preoperative clinical indicators were used to predict ER. Notably, clinicopathological factors may influence recurrence differently based on the different timepoint definitions for ER. A study that used a timepoint of 9 months^[17]^ identified four preoperative independent risk factors for ER, including elevated CA19-9 levels, elevated CA125 levels, tumor size, and regional lymph node enlargement on CT. Furthermore, Groot et al^[7]^ discovered that the preoperative risk factors for ER included a CACI ≥ 4, CA 19-9 level > 210 U/mL, and tumor size > 3.0 cm on CT. In our study, the preoperative risk factors for ER included a CACI ≥ 4, tumor size > 3.0 cm on CT, clinical symptoms, CA 19-9 level > 181.3 U/mL, and CEA level > 6.01. Taken together, CA 19-9 appears to be the most studied and established tumor marker for PDAC.

Multiple studies^[7,15,17,20–22]^ have reported an association between elevated preoperative CA 19-9 levels and post-pancreatectomy ER. Notably, proposed thresholds have varied from 100 to 529 U/mL, a variation that could be attributed to differences in the definitions of ER among studies.

In the current study, ROC curve analysis revealed that the optimal cutoff threshold for preoperative CA 19-9 levels in predicting ER was > 181.3 U/mL. The utility of preoperative CA 19-9 levels was fairly predictive, with an AUC, sensitivity, and specificity of 0.630, 0.572, and 0.654, respectively, highlighting the need to identify more accurate tumor markers for PDAC. Accordingly, we examined other biomarkers related to PDAC, including CEA and CA242, and found that only elevated CEA levels (>6.0 U/mL) were associated with ER.

Moreover, we found that the CACI was an independent risk factor for ER. Numerous studies have demonstrated that the CACI can be used to predict postoperative mortality in patients; moreover, the CACI has been extensively used to predict outcomes in patients with cancer^[12,22–26]^ and other medical conditions.^[27,28]^ Furthermore, high CACI scores are associated with prognosis in patients with PDAC.^[12,24]^ Notably, Groot et al^[7]^reported that a high CACI score was an independent preoperative risk factor for ER, which is consistent with our findings. This indicates that the CACI can be used to predict postoperative mortality and postoperative recurrence.

Additionally, we found that the preoperative clinical symptoms and tumor size on CT were independent risk factors for ER. The clinical symptoms include upper abdominal pain, lower back pain, and jaundice; further, only a few studies have investigated the correlation between clinical symptoms and ER. Barugola et al^[29]^ indicated that the preoperative duration of symptoms was a key factor for mortality within one year after PDAC resection. Notably, the prognosis of pancreatic cancer does not deteriorate with increased tumor size.^[30]^

After curative-intent surgery, many patients still experience ER. Further, selecting patients who may not benefit from upfront surgery is important. Therefore, we established a predictive model for PDAC based on five preoperative risk factors (CA 19-9, CEA, CACI, tumor size on CT, and symptoms).

A nomogram is a simple and intuitive representation of a mathematical model.^[31]^ The calibration of the model aligns with the agreement between predicted and observed outcomes of ER after pancreatectomy (Fig. 2A). Preoperative stratification of the risk of ER using our prediction model could inform therapeutic strategies.

This study has several limitations. First, this was a retrospective study, inevitably leading to selection bias. Second, adjuvant chemotherapy is an independent risk factor for early recurrence of pancreatic cancer after surgery, an important factor influencing our predictive model. Finally, we included a limited number of preoperative factors; therefore, the specificity and sensitivity of the model may be further improved by including other factors.

In conclusion, we identified independent preoperative predictors of ER after PDAC resection, including a CACI ≥ 4, tumor size > 3.0 cm on CT, clinical symptoms, CA 19-9 level > 181.3 U/mL, and CEA level > 6.01. A predictive model established using the aforementioned preoperative factors effectively identified patients at a high risk of ER and those who might not benefit from upfront surgery.

## Acknowledgments

We thank Tianxing Zhou, MD and Yongjie Xie, MD from the Department of Pancreatic Cancer at Tianjin Medical University Cancer Institute and Hospital, National Clinical Research Center for Cancer, Key Laboratory of Cancer Prevention and Therapy and Tianjin’s Clinical Research Center for Cancer. We would also like to thank Editage (www.editage.cn) for English language editing.

## Author contributions

**Data curation:** Quan Man, Huifang Pang, Yuexiang Liang, Shaofei Chang.

**Formal analysis:** Quan Man, Huifang Pang, Junjin Wang, Song Gao.

**Funding acquisition:** Quan Man, Huifang Pang.

**Investigation:** Quan Man, Huifang Pang, Yuexiang Liang, Song Gao.

**Methodology:** Quan Man, Yuexiang Liang, Shaofei Chang, Junjin Wang.

**Writing – original draft:** Quan Man.

**Writing – review & editing:** Quan Man, Song Gao.
